# Cadmium, Chromium, and Cobalt in the Organs of *Glyceria maxima* and Bottom Sediments of the Pisa River and Its Tributaries (Poland)

**DOI:** 10.3390/ijerph181910193

**Published:** 2021-09-28

**Authors:** Elżbieta Skorbiłowicz, Mirosław Skorbiłowicz, Urszula Tarasiuk, Magdalena Korzińska

**Affiliations:** Department of Technology in Environmental Engineering, Faculty of Civil Engineering and Environmental Sciences, Bialystok University of Technology, Wiejska 45E, 15-351 Bialystok, Poland; m.skorbilowicz@pb.edu.pl (M.S.); magdakorzinska@wp.pl (M.K.)

**Keywords:** potentially toxic elements (PTEs), *Glyceria maxima*, bottom sediments

## Abstract

The aim of the presented article was to determine whether human activity significantly influenced the enrichment of Cd, Co, and Cr, in river sediments and *Glyceria maxima*, in the basin of the Pisa River, an underdeveloped area in Poland. In this study, the content and spatial distribution of Cd, Cr, and Co in the organs underground and above ground, (sequentially: root, stem, leaf) of *Glyceria maxima* and bottom sediments of the Pisa River and its tributaries (Pisza Woda, Wincenta, Turośl and Skroda River) were analyzed. The Potentially toxic elements (PTEs) were determined by ASA method (Atomic Absorption Spectrometry). The results showed that the average PTEs contents in the river sediments occurred in the following descending order of Cd < Co < Cr. The highest values of the Igeo, CF coefficients, i.e., the greatest impact of anthropogenic activities on the water environment of the Pisa River and its tributaries, were found especially in the case of Cd. The research on the plant material has shown that the highest content of Cr and Co occurs in the roots, then in the stems, and the least in the leaves of *Glyceria maxima*. However, the amounts of Cd in the examined parts of *Glyceria maxima* had similar values. The content of Cd, Cr, and Co in the roots and above-ground parts exceeded the physiological values. *Glyceria maxima* can be used as a biological indicator material. Statistical analyzes showed the movement of PTEs in the sediment-root-stem-leaf system and identified the sources of PTEs, i.e., municipal wastewater treatment plants, the local food industry, and surface runoff.

## 1. Introduction

Rivers play a very important role in providing water for human consumption, but also in supplying agriculture and industry with it. Industrial plants, mining, agriculture, and large urban agglomerations generate a lot of pollutants, discharged in dissolved or suspended form into rivers, lowering the quality of water in rivers, which in turn may pose a threat to human health and life [1,2,3,4,5,6,7].

PTEs are one of the most toxic chemicals and their accumulation in the environment is one of the major global problems [8,9]. They have a longer biological half-life compared to other elements. Moreover, they are non-biodegradable, toxic, and persistent, thus posing a serious threat to aquatic ecosystems [10,11,12,13,14,15,16,17]. The sources of PTEs in the environment are mainly fertilizers, pesticides, untreated or only partially treated municipal and industrial sewage, leachate from mining areas, industrial waste, waste from ore metallurgy, and sewage sludge [11,18,19,20]. Various factors: physical, chemical, biological, and geological influence the PTEs found in rivers [21]. An important role in the migration of PTEs in surface waters is attributed to adsorption on suspended particles. Thanks to this process, PTEs are accumulated in the bottom sediments of water systems [22,23]. According to Zheng et al., 2008 about 90% of the PTEs in the aquatic environment are associated with sediments and suspended solids. Sediments are the main vector for the transport of toxic pollutants due to their large surface area, ensuring high sorption potential for dissolved components [24,25,26,27]. It should be emphasized that the level of water pollution may change rapidly depending on man-induced pressures and hydrodynamic processes, while bottom sediments are less active due to their properties [28]. According to Zhang et al., 2017, [29], bottom sediments provide information on the pollution of rivers, which is why their research is the basis for assessing the quality of aquatic ecosystems. Many macrophytes with high growth rates and various habitat, are also a good indicator of PTEs pollution in rivers [30,31]. They act as biofilters, accumulating PTEs from the surrounding environment. They take up PTEs from the water column and/or from sediments [32], due to their ability to absorb these elements by roots, stems, and leaves [33,34,35]. Aquatic plants are suitable for detecting environmental changes [36,37]. Many studies have demonstrated the ability of macrophytes to phytoremediate heavy metals [20,38,39,40,41,42,43,44,45]. Analysis of bottom sediments and biological samples helps to determine the overall content of PTEs and their impact on the aquatic environment [46]. Therefore, to assess the state of pollution of the aquatic environment, it is necessary to analyze samples of bottom sediments and aquatic plants [11]. The ability of macrophytes to remove PTEs may be important for the health of aquatic ecosystems, but also provides a tool to support traditional treatment systems [47].

The studies were aimed at determining whether human activity significantly influenced the enrichment of selected PTEs in river sediments and macrophytes in the catchment area of the Pisa River, an underdeveloped area in Poland. The area is hardly transformed by humans, there are no mines, no heavy industry, no large agglomerations, and the population density is still low. The outstanding natural value of this region contributed to the research into the ecosystem of the Pisa River and its tributaries. To the extent of our knowledge, there were no studies on the content of PTEs in bottom sediments and macrophytes. These are the first studies of this type. River water analyzes were performed at one point on the Pisa River (Morgowniki) and on the Turośl River (Potasie) under the National Environmental Monitoring in Poland. The choice of PTEs: Cd, Cr, and Co was dictated by the fact that agricultural soils and roadside soils in the studied area were slightly enriched, especially in Cd and Cr. The sites from which samples of bottom sediments and aquatic plants were usually collected located near bridges that run along communication routes. The guidelines for the quality of bottom sediments and macrophytes adopted in the research allowed for an accurate assessment of pollutants, as well as the estimation of the natural and anthropogenic contribution to enrichment of the studied rivers in PTEs.

The research was aimed at determining whether human activity significantly influenced the enrichment of selected PTEs in river sediments and macrophytes in the catchment area of the Pisa River, an underdeveloped area in Poland. The main objectives of the research were: (1) analysis of the content and spatial distribution of Cd, Cr, and Co in the organs (root, stem, leaf) of *Glyceria maxima* and bottom sediments of the Pisa River and its tributaries (Pisza Woda, Wincenta, Turośl, and Skroda); (2) assessment of the degree of pollution/enrichment of bottom sediments with PTEs using geochemical index (Igeo), CF, (pollution load index) PLI; (3) identification of local sources and determining factors of Cd, Cr, and Co in sediments and plant material by means of multivariate statistical analysis.

## 2. Materials and Methods

### 2.1. Study Area

The Pisa River is a right-bank tributary of the Narew and 80 km long. Pisa is a typically lowland river, it meanders strongly and creates numerous oxbow lakes. The river basin area is 4500 km^2^ and is located in the Kurpie Plain and the Piska Forest. Right-bank tributaries: Barłoga, Rybnica, Rudna, and the largest of them—Turośl. The left-bank tributaries of the Pisa River are: Szparka, Pisza Woda, Bogumiłka, Wincenta, and Skroda. The catchment area of the Pisa River is slightly changed by man and is characterized by a high degree of naturalness. About 70% of the western part of the Pisa catchment is covered with forests (Piska Forest). On the other hand, in the eastern part of the Pisa River basin, there is arable land. 

The main sources of pollution, i.e., PTEs, in the Pisa River catchment area result from the functioning and development of larger towns located within the basin. The quality of the environment of the Pisa River basin is influenced by: the lack or incomplete efficiency of the sewage system, sewage treatment plants, vehicle traffic, small industry (meat, wood, food), and agriculture (plant protection and fertilization), (Table 1) [48,49,50].

### 2.2. Sampling and Sample Preparation

**Figure 1:**
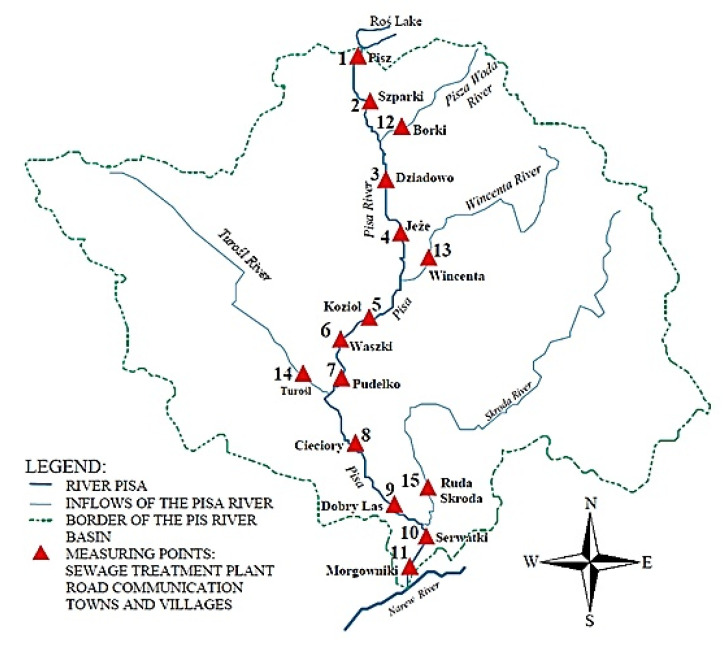
Research facility—the Pisa River and its tributaries.

Samples of bottom sediments and plants, i.e., *Glyceria maxima* (underground and above ground parts of the plants, in sequence: roots, stems, and leaves) were collected in August 2016 from 11 measurement points (Pisz, Szparki, Dziadowo, Jeże, Kozioł, Dragonflies, Pudełko, Cieciory, Dobry Las, Serwatki, Morgowniki) on the Pisa River and its four tributaries (Figure 1). *Glyceria maxima* was selected as a test (indicator) plant due: to occurrence in almost all studied rivers, being common throughout the country, good penetration of sediments—it has a crawling system of rhizomes and long runners, being characterized by a large number of occurrences and easy collection of plant samples from rivers, *Glyceria maxima* accumulates large amounts of elements, chemical analyzes are easier to interpret, and the analysis of these plants allows to recreate spatial and temporal differences in the concentration of elements in the aquatic environment and to determine the sources of pollution. The location of the measurement points has been selected in terms of the sources of pollution in the catchment area of the Pisa River. Samples of bottom sediments were collected in the coastal zone, where the sedimentation process of the suspended material and the concentration of PTEs take place. At each of the designated measurement points, samples of bottom sediments were taken from a depth of 5–10 cm from under the water surface. The bottom sediment samples were mixed, which allowed to obtain one representative test sample weighing about 1 kg [51]. The selected plant growing at each test site (*Glyceria maxima)* was collected from the same measurement points as the samples of bottom sediment. 

Samples for the analysis of aquatic plants were created from combining several individual macrophytes. *Glyceria maxima* is a species quite common in Poland. It also occurs in Europe and Asia [52].

### 2.3. Analytical Procedures

After collection, the bottom sediment samples were dried and stored. Next the bottom sediment samples were sieved through a 0.2 mm sieve prior to further analysis. The bottom sediments were mineralized with hydrochloric acid and nitric acid in a volume ratio of 3:1 in a closed CEM microwave system. All determinations were made in triplicate. After filtration, the samples were quantitatively transferred to 50 mL volumetric flasks. The content of PTEs Cd, Cr, and Co was determined by flame atomic absorption spectrometry on the AAS ICE 3500 Thermo Scientific spectrometer (Thermo Scientific Portable Analytical Instruments Inc., Tewksbury, MA, USA). The results of the sediment analyzes were verified using the NCS DC 73317a certified reference material for sediments. The calculated measurement error did not exceed 5% of the certified value.

Macrophytes transported to the laboratory were washed with tap water and distilled water, and then dried at 80 °C. The dried roots, stems, and leaves of *Glyceria maxima* were homogenized and digested with hydrochloric and nitric acid at a volume ratio of 3:1 in a closed CEM microwave system. Cd, Cr, and Co content was determined by flame atomic absorption spectrometry on AAS ICE 3500 Thermo Scientific spectrometer. The measurement error of the analysis was determined by comparing the obtained results with the characteristics of the mixture of grasses, ERM–CD281, and strawberry leaves, LGC7162. The calculated measurement error did not exceed 5% of the certified value. 

The results of the content of Cd, Cr, and Co presented in relation to the dry weight of plants and compared with the literature data. The physiological standard of PTEs content in plants was given according to the data of Kabat-Pendias and Pendias (2001), [53]. The bioconcentration factor were calculated as the ratio of Cd, Cr, and Co content in the plant root to the PTEs content in the bottom sediment. The translocation factor was calculated as the quotient of the content of Cd, Cr, and Co in the roots and stems as well as in the root and leaf of the examined macrophyte.

### 2.4. Assessment of Bottom Sediments Pollution Degree

In the interpretation of river sediment, contamination plays an important role selection of the geochemical background. The geochemical background is the concentration of particular chemical compounds or elements naturally occurring in the environment. The basic role of the geochemical background in environmental research is to determine whether the studied area is subject to anthropogenic impacts, or whether we are dealing with the effects of contamination, contamination, or enrichment. Many researchers, e.g., Islam et al., 2015, [54], used the mean content of an element in the earth’s crust proposed by Turekiana and Wedephola, 1961, [55]. PTEs content were also compared with the “globally” defined geochemical background [55] and with the value of the geochemical background determined locally for Polish sediments proposed by Bojakowska and Sokołowska, 1998, [56], (Table 2). The sediment contamination level was also used to assess the sediment quality of the Pisa River, using the Igeo, CF, and the PLI. 

The geochemical index (Igeo) was calculated using the following formula [57]:

(1) $$I_{\mathrm{geo}}=\log_{2}\left(\frac{C_{m}}{1.5\mathrm{GM}}\right),$$

where:

GM—geochemical background (mg∙kg^−1^),

C_m_—content of analyzed PTEs (mg∙kg^−1^), [55].

The values of the geoaccumulation index (Igeo) are divided into seven classes, i.e., uncontaminated sediments class 0 (Igeo ≤ 0), slightly polluted sediments class 1 (0 < Igeo < 1), moderately polluted sediments class 2 (1 < Igeo < 2), moderately contaminated sediments class 3 (2 < Igeo < 3), heavily polluted sediments class 4 (3 < Igeo < 4), heavily polluted sediments class 5 (4 < Igeo <5), and extremely polluted sediments class 6 (Igeo ≥ 5).

The CF coefficient was calculated as the ratio of the content of the tested PTEs to the background value [55] obtained in the sediments: 

CF_PTEs_ = C_PTEs_/C_tło_. (2)

On the other hand, to assess the degree of pollution, we used the four-level scale proposed for the CF coefficient: low degree (CF < 1), moderate degree (1 ≤ CF < 3), high degree (3 ≤ CF < 6), and very high degree (CF ≥ 6) [58].

The sediment quality was also determined by calculating the PLI (pollution load index) for the elements Cd, Cr and Co, which is a comprehensive measure of pollution by more than one element. PLI is an experimental formula developed by [59]:

PLI = (CF1 × CF1 × CF1 × ... × CFn)1/n,(3)

where n is the number of elements specified in the samples. The empirical index provides simple comparisons of the average PTEs contamination at different soil sampling sites. PLI value = 0 indicates excellence PLI < 1 indicates no impurities and PLI > 1 is an impurity [54].

### 2.5. Statistical Analysis

Descriptive statistics were used to explain the PTEs content in bottom sediments and macrophytes. Prior to analysis, the Shapiro-Wilk normality test was used to assess whether the original data met the requirements of a normal distribution. The Box-Cox transformation was performed for that part of the data that did not meet the normal distribution. The results were considered statistically significant with the probability of making an error *p* < 0.05. Pearson’s correlation coefficient (parametric test) was used to measure the interdependencies between two PTEs. Pearson’s correlation coefficients of relations between elements provide valuable information on sources in the geoenvironment [60]. Hierarchical cluster analysis (HCA) and factor analysis (FA) to investigate possible sources of PTEs (Cd, Cr, and Co) in aquatic plants and bottom sediments. In HCA analysis, the distance between clusters containing Cd, Cr, and Co was measured by the square of the Euclidean distance according to Ward’s method. Varimax with Kaiser normalization was used as the rotation method [61]. To assess the reliability of the FA, we used a measure of the Kaiser-Meyer-Olkina (KMO). Multivariate statistical analysis is widely used in environmental research, which provides an efficient way to reveal the relationship between multiple variables, and thus helps to understand the factors and processes influencing the migration of chemical components, as well as to identify their sources [62]. Statistical analyzes were performed using the STATISTICA software ver. 13.3.

## 3. Results and Discussion

### 3.1. PTEs Content in Bottom Sediments

The content of Cd, Cr, and Co in the samples of bottom sediments from the 11 designated research points on the Pisa River and 4 points on its tributaries is presented in Table 2. Average PTEs contents in river sediments occurred in the following decreasing order: Cd (0.37 mg·kg^−1^) < Co (3.36 mg·kg^−1^) < Cr (10.87 mg·kg^−1^). The lowest content was obtained for Cd, and it is largely dictated by the geochemical properties of this element. However, the Cd content of sediments is of great concern due to its high toxicity, and it has been described as one of the most unstable PTEs [63,64]. Much data is available, but gaps in the current state of knowledge make it difficult to assess the effects of cadmium on living organisms. This element, as well as some other PTEs, such as lead or mercury, play no biological role and are potentially highly toxic to plants, animals, and humans. [65]. The conducted research showed that Co and Cr are at the geochemical background level apart from Cd [55]. The maximum content of Cd was higher by 0.18 mgCd·kg^−1^ than the geochemical background, which indicates a slight enrichment of this element in the sediments. The source of Cd are vehicles emitting it together with dust from tire wear [66] and diesel engines [67]. This is due to the small diameter of the element’s particles, and thus its long residence time in the atmosphere. These particles are easily transported over long distances [68]. The combustion of hard coal should be distinguished among other significant sources of this element. Hard coal, used as a fuel for CHP plants, can be an important source of cadmium in the atmosphere [69]. This material is also an important raw material for heating homes. Its combustion causes the release of large amounts of dust pollutants containing various heavy metals, including Cd [70], and the elements released in this way may pollute aquatic ecosystems. It is estimated that crude oil has a lower cadmium content than coal, and natural gas does not contain significant amounts of this element and is considered to be a negligible source [71]. Cadmium can enter rivers through surface runoff from soil. High levels of this element in agricultural soil are achieved by repeated application of phosphorus and organic fertilizers [72]. Cadmium in the bottom sediment is highly mobile. It can be taken up by the plant root system and transported to the above-ground organs [53]. However, according to the classification used in Poland [56], the analysis of the results of the Cd, Cr, and Co content of the bottom sediment tests showed a slight excess of the geochemical background for Cr and Co (Table 2). According to Mohiuddin et al., 2012, [73], higher Cr levels may come from municipal wastewater. It is worth noting, however, that the greatest risk of chromium in sediment or soil is related to its hexavalent form, while trivalent Cr is relatively immobile, which causes lower risk of its presence [74]. The potential for Co transfer from soil or sediment to plant roots is rather low [75]. The average content of the Cd, Cr, and Co in the sediments of Pisa River and its tributaries is much lower than the average content of these PTEs in bottom sediments occurring in other rivers in Poland [51].

### 3.2. Assessment of Contamination with PTEs (I_geo_, PLI, CF)

**Figure 2:**
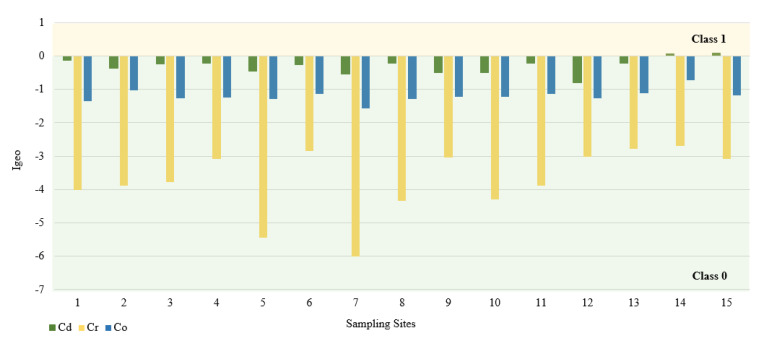
Igeo values for PTEs at different sampling sites.

**Figure 3:**
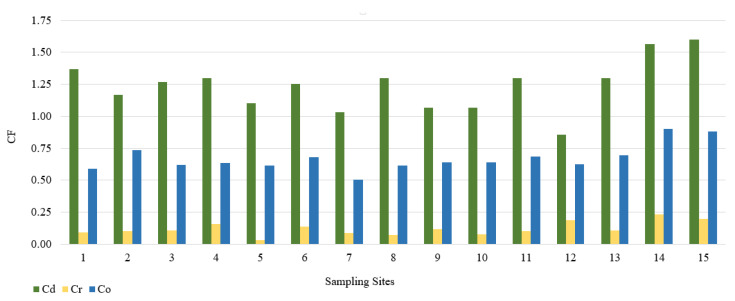
Value of the CF in bottom sediments of Pisa River and its tributaries.

In this study, we used the geoaccumulation coefficients Igeo, the pollution load index PLI and the pollution factor CF. The calculated value of the Igeo geocumulation index is presented in Figure 2. Among the tested PTEs, the mean Igeo values showed the following order: Cd (−0.3) > Co (−1.2) > Cr (−3.7). According to the Müller scale, the calculated results of the Igeo value showed that the sediments from 15 sampling points belong to class 0, and therefore are not contaminated with Cd, Co, and Cr. The highest Igeo values occurred in the case of Cd. Analyzing the maximum Igeo values for Cd (0.06 and 0.09), found at points 14 and 15 located on Turośl and Skroda rivers, indicates a slight enrichment in these PTEs. Cadmium is one of the highly toxic elements. Therefore, even at low concentrations, Cd may be harmful to living organisms. The main source of Cd may be treated wastewater discharged into the Turośl and Skroda Rivers. According to Fujita et al., 2014, [76] the use of phosphorus fertilizers and pesticides in agriculture can enrich river bottom sediments in Cd. The bottom sediments collected on the Pisa River and its tributaries can be considered slightly unpolluted with Co and Cr, the highest Igeo values occurred at the collection point 14—Turośl River (Co-Igeo (−0.73) and Cr-Igeo (−2.69)), (Figure 2). The mean value of the contamination factor CF was in the following order Cd (1.24) > Co (0.88) > Cr (0.13), (Figure 3). The pollution coefficient (CF) value for Cr and Co showed a low degree of pollution (CF < 1). In contrast, Cd showed moderate (1 ≤ CF < 3). Trace elements with high CF values, which usually have a short retention time and high mobility in sediments, which poses a high risk to the aquatic environment and the ecosystem [77].

**Figure 4:**
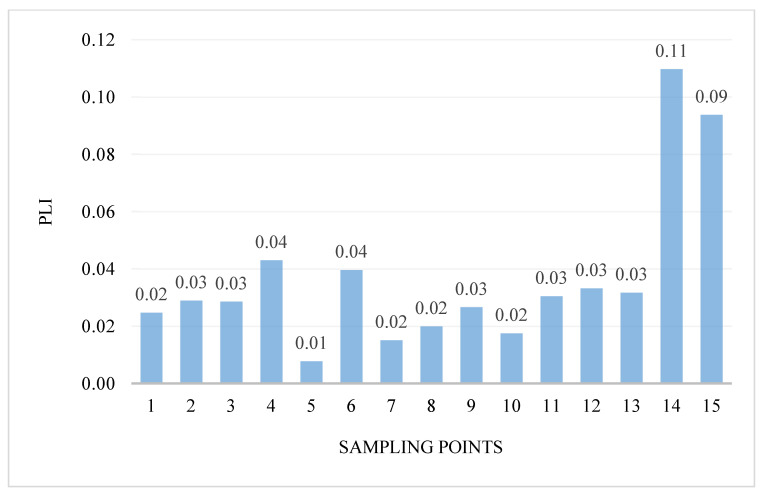
Value of the PLI in bottom sediments of Pisa River and its tributaries.

The intensity of pollution and its variability in the areas were determined using the pollution load index PLI. PLI values ranged from 0.01 to 0.11 (Figure 4). Generally, the PLI values indicate the highest pollution at two research points: 14—the Turośl River (0.11) and in point 15—the Skroda River (0.09), this indicates the contribution of trace PTEs from anthropogenic sources. However, in none of the examined points was the value of the PLI coefficient above 1.

### 3.3. PTEs Content in Glyceria Maxima

**Figure 5:**
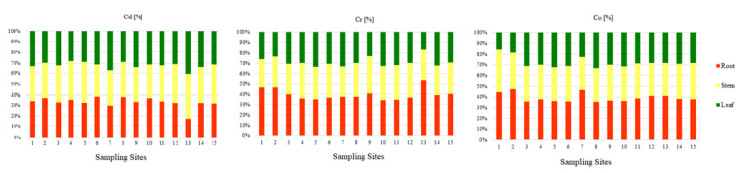
Percentage of trace elements (Cd, Cr, Co) in individual parts of aquatic plants (root, stem, leaf) collected in the Pisa River and its tributaries.

The ranges of the average content of the examined elements in the roots, stems, and leaves of *Glyceria maxima* collected in the Pisa River and its four tributaries are presented in Table 2. On the basis of the obtained results, it was observed that the greatest amount of Cr and Co was in the roots (9.40 mgCr·kg^−1^; 3.46 mgCo·kg^−1^) than in the stems (7.73 mgCr·kg^−1^; 2.85 mgCo·kg^−1^), and the least in leaves (7.17 mgCr·kg^−1^; 2.48 mgCo·kg^−1^). The percentage of Cr and Co in the individual parts of *Glyceria maxima* (Figure 5) is as follows: about 40% is on the roots, about 35% on the stems, and the least amount of PTEs is in the leaves (about 25%). According to Baldantoni et al. (2004) [78], the high content of PTEs in the underground parts of plants and the low content in the above-ground parts of plants indicate that the bottom sediments are the main source of the tested PTEs and only a small part is transferred to the stems and leaves. The Cd content was different in the case of stems (0.37 mgCd·kg^−1^), followed by roots (0.35 mgCd·kg^−1^), and the lowest in leaves (0.34 mgCd·kg^−1.^ It should be noted, however, that the amount of Cd in the tested parts of *Glyceria maxima* had similar values, the differences ranging from 0.01 mgCd·kg^−1^ to 0.03 mgCd·kg^−1^. The percentage of Cd shows an almost equal division between the tested parts in the plant (Figure 5). It was shown that the contents of Cd, Cr, and Co in the roots and above-ground parts exceeded the physiological values presented by Kabata-Pendias and Pendias (2001) [53], which proves the contamination of the Pisa River and its tributaries with these PTEs (Table 2). According to Kabata-Pendias and Pendias (2001) [53], Cd, it is very easily absorbed by the roots and leaves of plants. Transport of Cd in the plant is easy, but with its more intensive uptake, it accumulates in the roots. Cadmium is toxic to plants, both directly and indirectly through interaction with other PTEs. Chromium plays no known biological role in plant physiology. Therefore, it is concluded that the toxicity of Cr influences the growth of plants and inhibits their basic metabolic processes [79]. On the other hand, Zayed et al., 1998, [80], claim that the distribution and displacement of Cr in plants depends on the plant species, the degree of oxidation of Cr ions, as well as its concentration in the environment. Kabata-Pendias and Pendias, 2001 [53], state that Cr belongs to the elements passively absorbed by plants, and its uptake and transport is closely related to the presence of Fe. Compared to other PTEs, the mobility of Cr in plant roots is low [81]. While there is evidence of a beneficial effect of low Co concentrations on plant growth, it has not been confirmed whether the presence of Co is essential in plants [82]. In higher plants, cobalt is transported to tissues by active and passive transport in a manner similar to iron uptake, therefore, with the same form of cation uptake, antagonism between cobalt and other elements is visible in plants [83]. Absorption of Co is inhibited by high concentrations of Ca, Fe, and Mn [53]. Bonanno, 2011, [79] claims that macrophytes accumulate microelements due to their constant contact with water and have been used as a biological indicator material for years.

### 3.4. BF and TF Coefficient

In order to assess the transport of Cd, Cr, and Co from the bottom sediment to the macrophyte roots, the bioaccumulation factor (BF) was calculated, which is presented in Table 3. The obtained mean BF value increased in the order: Co > Cd > Cr and was below one for Cd (0.94) and Cr (0.86), but in the case of Co the result was 1. Similar results were obtained in their studies by [84].

The translocation factor (TF) provides information about the transport of PTEs in the plant. The values of the TF coefficient are illustrated by the mobility of Cd, Cr, and Co in the root-stem, root-leaf relationship. TF values > 1 indicate a high degree of PTEs movement in *Glyceria maxima*. The obtained mean values of the coefficient are less than 1.0 in the case of Cr and Co. The mobility of tested Cr and Co differed in the analyzed parts (root, stem, and leaf) of *Glyceria maxima*, higher TF was noted between the roots and the stem (0.82–Cr, 0.85–Co). However, lower values were obtained between the root and the leaf (0.76–Cr, 0.74–Co). Mean values of TF were recorded at the unity level, for Cd between the root and stem (1.07) and the root and leaf (0.97), (Table 3). This means that *Glyceria maxima* does not effectively transfer PTEs from the root to the aerial parts. The plant accumulates PTEs in underground organs better than in above-ground organs. The high concentrations of PTEs observed in the roots suggest some level of tolerance to PTEs, through the existence of protective mechanisms limiting the transfer of these toxic compounds from the roots to the stems and leaves [85]. The high concentration of Cd, Cr and Co found in the roots can be mainly attributed to the fact that the absorption process takes place through the roots. In addition, plant physiology also plays an important role in excluding some PTEs, the presence of which is not essential in the plant, and thus protect the above-ground parts (stem, leaf) of the plant [86].

### 3.5. Spatial Distribution of PTEs Content

**Figure 6:**
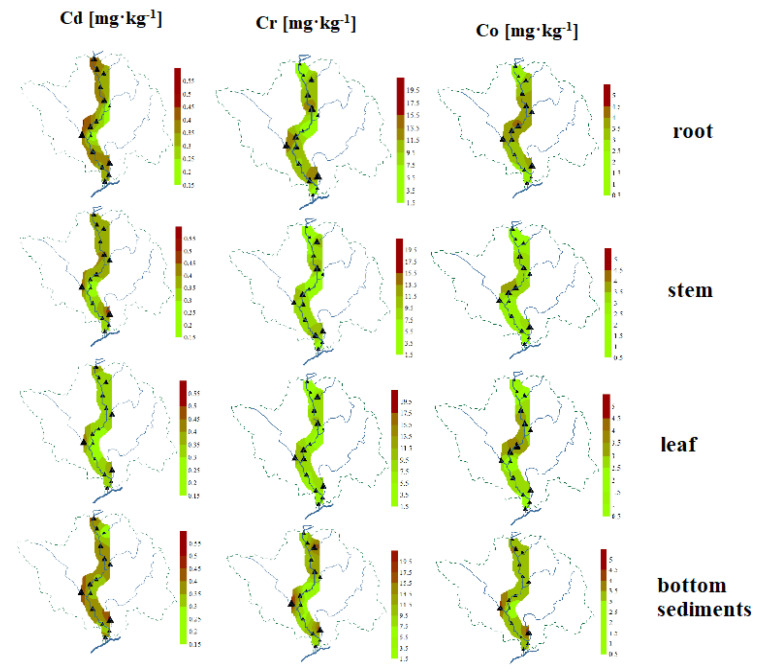
Spatial distribution of PTEs (Cd, Cr, Co) in roots, stems, and leaves of aquatic plants and bottom sediments on the Pisa River and its tributaries.

The study of the spatial distribution of Cd, Cr, and Co in bottom sediments and *Glyceria maxima* (leaves, stems, roots) is helpful in identifying places with increased PTEs content (Figure 6). They show similarities, although with different details, and some general regularities can be observed. The highest accumulation of these PTEs occurred at points located on tributaries, in particular on the Turośl and Skroda rivers. It seems that similar phenomena affect the spatial distribution of Cd, Co, and Cr, which was confirmed by the correlation coefficients between PTEs. This is due to the impact of wastewater treatment plants located in the vicinity of the above-mentioned points and the impact of wastewater from the food industry. A study Zhang et. al. (2010) [87] claims that the presence of PTEs in the river may be associated with continuous discharge of most municipal wastewater. The discharge of municipal and industrial wastewater directly into river systems is an important problem for aquatic environments and poses a potential threat to human health [88].

### 3.6. PTEs Content Results in Rivers around the World

The obtained PTEs contents in bottom sediments and macrophyte (*Glyceria maxima*) taken from the Pisa River and its tributaries were compared with the PTEs contents in sediments and aquatic plants from rivers in other parts of the world (Table 4 and Table 5). However, it should be remembered that each river is unique. The content of PTEs in sediments and aquatic plants is the result of physical, chemical, biological, and geomorphological processes occurring both in the riverbed and in the catchment area, in which climate also plays a significant role. Moreover, the results are often obtained by different research methods, so such a comparison can only be approximate. In the case of Cd content in bottom sediments, the results were lower or similar to those obtained by the authors, as shown in Table 4, but slightly higher values were presented by Bonanno and Giudice (2010) [79] and Samecka and Kempers (2007) [89]. The content of Cr in the sediments of Pisa and its tributaries significantly differed from the values obtained by other authors (5.78 ± 0.47 to 237.40 ± 233.60 mgCr·kg^−1^) The exception was the Bug River, in which the content of this element was very similar to the concentration of Cr in the sediments of the rivers studied. It is similar in the case of Co, where the most similar result for this element was obtained for the Bug River [90]. Other results presented by other authors were much higher. In the case of the content of the tested PTEs (Cd, Cr, and Co) in the plant material, all the results obtained by other authors were higher than the values obtained in this study (Table 4). An exception is the Cr submitted by Bonanno and Giudice (2010) [79] for *Phragmites australis.*

### 3.7. Identification of Pollution Sources Using Statistical Analyzes

**Figure 7:**
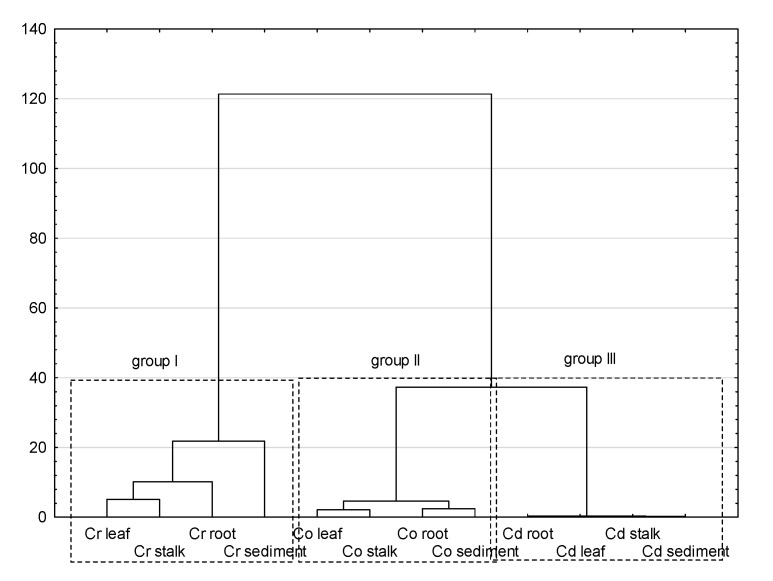
Hierarchical cluster analysis.

The process of statistical inference began with the basic analysis of the content of Cd, Cr, and Co in bottom sediments and plant material of the Pisa River and its tributaries. Coefficients of variation are used to reflect the variability of the analyzed items, the greater the CV value, the greater the variability. As shown in Table 2, CV values of all PTEs were consistent with the Cr > Cd > Co order in the case of sediment, roots, and stems, while the series in *Glyceria maxima* leaves was slightly different: Cr > Co > Cd. The high CV value for Cr and Cd indicates high variability and suggests that the sources of PTEs may come from external factors within the analyzed catchment. The analyzes of infrastructure in the Pisa direct catchment indicate that municipal wastewater treatment plants located in Kolno, Stawiski, Grabowo, and Turośl discharging treated wastewater into the water in the studied area, may be the sources of Cr, Cd, and Co, to a lesser extent. The source of the tested PTEs may also be sewage from the food industry. According to Kabata-Pendias, 2007 [93], both industrial and municipal wastewater treatment plants discharge significant amounts of Cr and Cd. It is also possible that runoff from the catchment area and roads may be sources of Cr and Cd [66]. Table 6 presents the Pearson correlation coefficients as the strength of the correlation between the studied elements. Before the decision to apply the Pearson coefficient, the Shapiro-Wilk version of the normality test was performed, which showed normal distributions of the analyzed data groups. While analyzing the Pearson correlation, a number of correlations were found between the content of PTEs in bottom sediments and individual parts of the plant, i.e., roots, stems, and leaves. Correlations represented by high Pearson coefficients indicate the mechanisms of PTEs relocation in the sediment-root-stem-leaf system. To confirm the above analyzes, we carried out a multivariate cluster analysis in the Ward’s version presented in Figure 7. In Figure 7, three distinct clusters (groups) were formed, depending on the content of individual PTEs in the tested elements. Group I, representing the content of Cr, was classified as an isolated group compared to groups II and III. The obtained classification allows us to state that the content of Cr is slightly similar to the content of Cd and Co, which in fact constitute one cluster. For further data exploration, we used factor analysis (FA), as shown in Table 7. Factor analysis showed two factors. The first factor F1 was correlated with Cr (root, stem, leaf) and Co (root, stem, leaf). Factor F1 explains as much as 50% of the variance, which indicates the dominant processes of Cr and Co enrichment in the analyzed system. The second factor explains only 22% of the variance (variation) and is correlated with Cd (sediment, stem, leaf) and Co (sediment). In total, the two factors detected explain 72% of the variance.

## 4. Conclusions

The presented article serves to establish whether human activity significantly influenced the enrichment of Cd, Co, and Cr in river sediments and *Glyceria maxima* in the catchment area of the Pisa River, an underdeveloped area in Poland. Average PTEs contents in river sediments occurred in the following descending order: Cd < Co < Cr. The studied coefficients (Igeo, CF, and PLI) can be considered the best tool to assess PTEs pollution, and they helped to determine the degree of anthropogenic impact on bottom sediments. The highest values of the calculated coefficients (Igeo, CF), i.e., the greatest impact of human activity on the environment of the Pisa River and its tributaries, were found especially in the case of Cd. The research on the plant material has shown that the highest levels of Cr and Co are found in the roots, than in the stems, and the lowest in the leaves of *Glyceria maxima*. However, the amounts of Cd in the examined parts of *Glyceria maxima* had similar values. The content of Cd, Cr, and Co in the roots and above-ground parts exceeded the physiological values. The mean BF value increased in the following order: Co > Cd > Cr. The obtained average values of the TF coefficient are less than 1.0 for Cr and Co, and about 1.0 for Cd. This means that *Glyceria maxima* does not effectively transfer PTEs from the roots to the aerial parts. *Glyceria maxima* is a plant commonly found in lowland rivers in north-eastern Poland and can be used as a biological indicator material. The contents of Cd and Cr, and to a lesser extent, Co, in sediments and plant material were largely related to human activities. The spatial distribution of the analyzed PTEs showed that the most PTEs were present in sediments and plant material in the tributaries of the Pisa in the Skroda and Turośl rivers. Statistical analyzes identified the sources of PTEs, such as municipal sewage treatment plants, the local food industry, surface runoff, and communication.

## Figures and Tables

**Table 1:** Measurement points located on the Pisa River and its tributaries and sources of pollution.

River	Place Measuring Points	No	Sources of Pollution
Pisa (82 km)	Pisz	1	urbanized area–the city of Pisz, wastewater from sprinkling–plywood factory
	Szparki/Niedźwiedzie	2	sewage treatment plant–municipal sewage treatment plant in Pisz
	Dziadowo	3	agro–forest area
	Jeże	4	agro–forest area
	Kozioł	5	agro–forest area
	Waszki	6	village of 167 inhabitants, arable land
	Pudełko	7	agro–forest area, the voivodeship road No. 647
	Cieciory	8	agro–forest area
	Dobry Las	9	agro–forest area
	Serwatki	10	agro–forest area
	Morgowniki	11	agro–forest area, the provincial road No. 648
*The tributaries of the Pisa River*			
Pisza Woda (12.8 km)	Borki	12	agro–forest area
Wincenta (25 km)	Wincenta	13	agro–forest area
canal Turośl	Turośl	14	agro–forest area, treated sewage–municipal sewage treatment plant in Turośl
Skroda (right) (50 km)	Ruda Skroda	15	agro–forest area, treated sewage–municipal sewage treatment plant in Kolno, KURPIANKA dairy production plant in Kolno, municipal sewage treatment plants in Grabów and Stawiaki

**Table 2:** Basic statistical data (*n*—11) of the content of studied indicators in bottom sediments and *Glyceria maxima* (root, stem, and leaf) in Pisa River and its tributaries.

Element mg·kg^−1^ DM	Cd	Cr	Co
Root			
±min–max	0.16–0.46	5.68–13.90	2.36–4.10
mean ± SD	0.35 ± 0.08	9.40 ± 3.12	3.46 ± 0.57
coefficient of variation [%]	24.30	33.23	16.98
Shapiro Wilk test (W)	0.87	0.87	0.94
Stem			
min–max	0.21–0.52	3.18–12.80	1.76–3.70
mean ± SD	0.37 ± 0.07	7.73 ± 3.02	2.85 ± 0.59
coefficient of variation [%]	20.86	38.99	20.71
Shapiro Wilk test (W)	0.93	0.94	0.94
Leaf			
min–max	0.23–0.48	1.77–11.10	0.95–3.70
mean ± SD	0.34 ± 0.06	7.17 ± 2.94	2.48 ± 0.81
coefficient of variation [%]	18.94	41.02	32.58
Shapiro Wilk test (W)	0.92	0.94	0.94
Bottom sediments			
min–max	0.26–0.48	3.10–20.96	2.53–4.51
mean ± SD	0.37 ± 0.06	10.87 ± 4.77	3.36 ± 0.52
coefficient of variation [%]	16.22	43.83	15.42
Shapiro Wilk test (W)	0.96	0.94	0.86
pH min–max	6.13–8.00		
MO [%] min–max	0.18–3.68		
Natural levels in plants	0.05–0.2 ^A^	0.02–0.5 ^A^	0.01–0.8 ^A^
Geochemical background	0.3 ^B^, 0.5 ^C^	90 ^B^, 5 ^C^	19 ^B^, 2 ^C^

^A^ Kabata-Pendias and Pendias (2001) [53], ^B^ Turekiana, Wedephola (1961) [55], ^C^ Bojakowska, Sokołowska (1998) [56].

**Table 3:** Transfer factor of PTEs from roots to other organs *Glyceria maxima* of from Pisa River (mean) and its tributaries.

Element	Root/Sediment	Stem/Root	Leaf/Root
Cd	0.94	1.07	0.97
Cr	0.86	0.82	0.76
Co	1.00	0.85	0.74

**Table 4:** Comparison of the results of PTEs content in bottom sediments for other rivers of the world.

Research Object	Avarage Content ± Standard Deviation (mg·kg^−1^)			Literature
	Cd	Cr	Co	
Pisa River, Poland	0.37 ± 0.06	10.87 ± 4.60	3.36 ± 0.50	This study
Rivers in Poland	2.80	18.00	ND	Lis and Pasieczna (1995) [51]
Oława River, Poland	0.35 ± 0.12	19.40 ± 8.20	ND	Samecka-Cymerman, Kempers, (2007) [89]
Piława River, Poland	0.85 ± 0.44	48.60 ± 20.00	ND	
Bug River, Poland	0.50 ± 0.24	10.50 ± 6.35	4.40 ± 1.04	Skorbiłowicz 2014 [90]
Meridionale River, Italy	0.66 ± 0.04	40.10 ± 2.33	ND	Bonanno, Guidice 2010 [79]
Xixiang River, China	ND	89.58 ± 43.99	ND	Liu et al. 2019 [91]
Alaro River, Nigeria	0.47 ± 0.03	5.78 ± 0.47	9.60 ± 1.60	Ipeaiyeda, Onianwa 2017 [92]

**Table 5:** Comparison of the results of PTEs content in macrophytes for other rivers of the world.

Research Object	Plant Species		Avarage Content ± Standard Deviation (mg·kg^−1^)			Literature
			Cd	Cr	Co	
Pisa River, Poland	*Glyceria maxima*	Root	0.35 ± 0.08	9.40 ± 3.02	3.36 ± 0.55	This study
		Stem	0.37 ± 0.07	7.73 ± 2.91	2.85 ± 0.57	
		Leaf	0.34 ± 0.06	7.17 ± 2.84	2.48 ± 0.78	
Ołobok River, Poland	*Elodea canadensis*		1.50 ± 0.90	23.00 ± 15.00	4.00 ± 2.20	Samecka-Cymerman, Kempers 2007 [89]
Piława River, Poland			1.50 ± 0.40	55.00 ± 21.00	23.40 ± 3.50	
Meridionale River, Italy	*Phragmites australis*	Root	1.13 ± 0.08	6.97 ± 0.19	ND	Bonanno, Giudice 2010 [79]
		Stem	0.68 ± 0.06	0.40 ± 0.04	ND	
		Leaf	1.05 ± 0.10	0.69 ± 0.04	ND	

**Table 6:** Pearson’s correlation coefficient.

	Cd Sediment	Cd Root	Cd Stem	Cd Leaf	Cr Sediment	Cr Root	Cr Stem	Cr Leaf	Co Sediment	Co Root	Co Stem	Co Leaf
**Cd Sediment**	1.00											
**Cd Root**	0.48	1.00										
**Cd Stem**	0.68	0.58	1.00									
**Cd Leaf**	0.75	0.52	0.87	1.00								
**Cr Sediment**	0.41	0.44	0.65	0.68	1.00							
**Cr Root**	0.22	0.31	0.30	0.24	0.77	1.00						
**Cr Stem**	0.03	0.30	0.16	0.04	0.63	0.93	1.00					
**Cr Leaf**	0.12	0.33	0.18	0.08	0.63	0.90	0.91	1.00				
**Co Sediment**	0.70	0.53	0.79	0.81	0.70	0.34	0.18	0.21	1.00			
**Co Root**	0.38	0.03	0.44	0.29	0.39	0.52	0.44	0.54	0.28	1.00		
**Co Stem**	0.50	0.33	0.54	0.39	0.36	0.52	0.48	0.53	0.35	0.89	1.00	
**Co Leaf**	0.23	0.14	0.31	0.11	0.20	0.44	0.51	0.52	0.24	0.76	0.86	1.00

**Table 7:** Factor analysis.

	Factor 1	Factor 2
Cd sediment	0.10	0.83
Cd root	0.19	0.63
Cd stem	0.20	0.91
Cd leaf	0.04	0.95
Cr sediment	0.54	0.62
Cr root	0.88	0.19
Cr stem	0.91	0.00
Cr leaf	0.92	0.05
Co sediment	0.16	0.89
Co root	0.73	0.26
Co stem	0.72	0.39
Co leaf	0.74	0.13
%VAR.	50.00	22.00
